# Editorial

**Published:** 1866-05

**Authors:** 


### Editorial.

### CONVENTION OF REPRESENTATIVES FROM MEDICAL COLLEGES OF THE WEST.

In response to a call of the Faculty of the Ohio Medical College for a meeting of delegates from the Medical Colleges of the West, for the purpose of agreeing upon a more uniform rate of lecture fees, the several delegates assembled in the faculty-room of the Ohio Medical College, in Cincinnati, at 10 o'clock A.M., of April 24th, 1866.

The meeting was called to order by Prof. M. B. Wright, of Cincinnati, who, after briefly stating the objects for which the meeting had been called, moved that Prof. N. S. Davis, of Chicago, be Chairman of the Convention. The motion was carried unanimously, and Prof. Davis took the Chair.

On motion of Prof. Mendenhall, of Cincinnati, Prof. Gustav C. G. Weber, of Cleveland, was appointed Secretary.

The members present, were as follows:—

M. B. Wright, from the Medical College of Ohio.

Geo. Mendenhall, from the Miami Medical College.

N. S. Davis, from the Chicago Medical College.

Francis Carter, from the Starling Medical College.

Gustav C. G. Weber, from the Charity Hospital Med. Col.

S. H. Douglas, from the Med. Dep't of University of Mich.

T. M. Holloway, from the Medical Department of University of Louisville.

Prof. Wright presented letters from the Deans of the St. Louis Medical College, the Nashville University, and the Medical Department of the Iowa University, expressing a concurrence in the object of the meeting, and an assurance that the schools they represented would sanction such uniform and advanced rate of fees as should be agreed upon by the Convention. The colleges not represented either by delegates or letters were, Rush Medical College, at Chicago; Cleveland Medi-College, at Cleveland; Cincinnati College of Medicine and Surgery, at Cincinnati; Medical Department of Humboldt University, at St. Louis; and the Kentucky School of Medicine, at Louisville.

To elicit the wishes of the Convention, Prof. Wright offered a resolution declaring that the sum of $105 should be adopted as a uniform rate of lecture fees by the Medical Colleges in the West. A very free and pleasant interchange of views followed, in which all the members participated, and during which it was made apparent that the immediate adoption of such a standard was impracticable, mainly on account of the peculiar position of the Medical Department of the University of Michigan.

After a recess for dinner, the Convention re-assembled at 4 o'clock P.M., when Prof. Davis offered the following preamble and resolutions, as a substitute for the resolution offered by Prof. Wright. The latter accepted the proposition, and after another free interchange of views in relation to the whole subject of Medical College organizations, as well as fees, they were unanimously adopted, as follows:—

*Whereas*, the cause of medical education requires the establishment and maintainance of permanent colleges, with all the necessary means for illustration and practical instruction, as well as competent teachers, thereby involving a large annual expenditure of money, therefore,

1st, *Resolved*, That a reasonable demand for lecture fees is required by the best interests both of the colleges and those who patronize them.

2d, *Resolved,* That competition among Medical Colleges, to be beneficial to the profession and the cause of medical science, should be based *entirely* on the ability of those engaged as teachers, and the completeness of their curriculum, with the facilities for practical demonstrations accompanying it, and not on *mere pecuniary* differences in the cost of attendance; and, hence, the fees charged in all the Medical Colleges, in a given section of country, should be uniform, or so near uniform that the actual cost of attendance in the different colleges shall be practically equal.

3d, *Resolved,* That inasmuch as only a limited number of students can be properly accommodated or educated in any one college each year, any State which, with enlightened liberality, should so endow the medical department of its State University as to make education therein free, ought to so far regard the interests of the institutions of other States, as to limit the freedom of its instruction to the citizens of its own State.

4th, *Resolved,* That in the opinion of the college faculties here represented, the aggregate annual fees for instruction in each college should be not less than $105 for each student.

5th, *Resolved,* That a committee of three be appointed, to communicate the foregoing views to the faculties of the several medical colleges not here represented, and also to the Regents of the University of Michigan, with a view to the ultimate removal of such obstacles, legal or otherwise, as may be in the way of the voluntary adoption of the sum named in the 4th resolution, or some other sum near it, as a uniform standard of college fees; and to take such measures as they may deem necessary, and report to a future Convention called for that purpose.

6th, *Resolved,* That the colleges here represented would, in the opinion of the delegates present, be willing to lengthen their annual lecture terms to six months, if, by so doing, practical uniformity in the standard of fees could be fully secured.

On motion of Prof. Holloway, the Secretary was authorized to furnish a synopsis of the proceedings of this Convention to the different medical journals of the country for publication.

On motion of Prof. Carter, Professors M. B. Wright, of Cincinnati, T. M. Holloway, of Louisville, and N. S. Davis, of Chicago, were appointed the committee called for by the 5th resolution.

Prof. Carter also moved, that when this Convention adjourn, it be to such time and place as the committee just named may deem proper; which motion was adopted.

On motion of Prof. Holloway, the Convention then adj'd.

The meeting was certainly a very pleasant one; the discussions throughout were cordial, friendly, and sincere; and we think it constitutes the beginning of a movement which, if followed up in the same spirit, will lead to the speedy adoption of several measures of great importance, both to the Colleges and the profession at large.

[For the information of students interested in knowing the price of lecture tickets during the coming winter, we would state that the contemplated advance can only take place after *all the Western colleges* have agreed to it, and that such an agreement cannot, by any possibility, be arrived at under a year or two. There will, therefore, be no rise in the prices next winter, except, perhaps, in a few schools whose rates are now below the average. E. A.]

---

TRICHINA.—The aversion to pork, produced by the agitation of the subject of trichiniasis, has induced the Chicago Academy of Sciences to appoint a committee to investigate the subject, with a view to ascertain the exact state of facts, in order that the public may know whether the trichina exists in this region or not, and if it does, whether the consumers of pork can, in any way, be assured of safety in the use of this important staple. The present state of uncertainty in the public mind is injurious to various interests.

The committee commenced by obtaining specimens of muscle from about 1300 different hogs, from all parts of the West, fed in various ways, and raised in all sorts of circumstances. These specimens were distributed among eight of the best microscopists of this city, for examination. The result showed that trichina, beyond all doubt, exists here, and probably has existed through all time wherever hogs have lived. Among the 1300 animals, 28 were found to contain the parasite, which is rather more than 1 animal in 50, or 2 per cent. Of these, only 3 or 4 contained them in dangerous quantities. The committee reports that the statement that trichinæ can endure a boiling heat without death, is false and absurd. By actual experiment, they are found to be always killed by a temperature of 150° Fah. It is proved, also, that salting and smoking for 10 days is fatal to them. A case of trichiniasis is reported at Detroit, Mich., in the *Medical Review*, in which a woman, lately arrived from Germany, died, having eaten the parasites before her arrival here. Two cases were also reported in the *Buffalo Medical Journal*, two or three years since, but with the exception of these three, the committee cannot learn of any deaths clearly traceable to trichiniasis in this country. In Germany, where so many deaths have occurred, the poor people have a habit of eating a large amount of raw pork, in which state the parasite is taken alive into the stomach of the patient; but in this country, pork is universally well cooked, even by the most indigent. This accounts for the fact, that though trichinæ are doubtless as ancient and universally distributed as the hogs themselves, and Americans have always made pork a very prominent article of diet, yet the disease is almost unknown this side of the water.

The conclusion is, that pork in a raw state, or not thoroughly cooked, is a dangerous food; but, when well cooked to the centre, is as absolutely safe as any other article of diet. It is proper to add, that trichinæ are found in a variety of animals besides swine, and that the same rule, as to thorough cooking, ought to be applied to all, whether fish, flesh, or fowl. Not only is trichina found widely distributed, but also the germ of the tapeworm may be found in a variety of the animals used for food. Beef and mutton are, probably, more free from parasitic dangers than any of the meats in common use.

---

CHOLERA. The cholera in New York is said to be still confined to Quarantine. In Halifax, the Health Officer, Dr. SLAYTER, is reported to have died of cholera, contracted while in attendance on the cholera-patients quarantined from the steamer England. The daily papers have telegrams, stating that the disease is raging at Key West, Florida, and give rumors of deaths from it in Washington and Cincinnati. On the whole, the appearances indicate that the disease is likely to pay another series of visits to all our chief towns. It behooves all, therefore, to be ready, and to instruct the public in all those hygienic measures adapted to limit the ravages of the prospective epidemic.

---

ILLINOIS STATE MEDICAL SOCIETY.—The next Annual Meeting of this Society will be held in *Decatur*, on the first Tuesday in June next. All local Medical Societies in the State are entitled to one delegate for every *five* resident members; every Medical College to two delegates; and every Public Hospital to one delegate. N. S. DAVIS, *Permanent Sec'y.*

---

NOTICE TO SUBSCRIBERS.—During the past few weeks, we have been sending *receipts* to all of our subscribers who have paid up to the end of this year in advance, and *bills*, showing the balance due, on all who have not so paid. We wish to get the books settled, and have every one of our patrons know the exact condition of his account. If any discover mistakes in the bills they receive, we hope they will promptly inform us, and we will take great pleasure in making all needed corrections.

---

BOOKS RECEIVED.—We have received the following new works:—

A MANUAL OF THE PRINCIPLES OF SURGERY, BASED ON PATHOLOGY. FOR STUDENTS. By WILLIAM CANNIFF, Licentiate of the Medical Board of Upper Canada, etc., etc., etc. Philadelphia: LINDSAY & BLAKISTON. 1866. Price $4.50.

DIARRHŒA AND CHOLERA; THEIR ORIGIN, CAUSE, AND CURE BY MEANS OF ICE. By JOHN CHAPMAN, M.D., etc., etc.

For sale by S. C. GRIGGS & Co., Lake St. Price 25 cents.

